# Supplementary material for: RgsA Attenuates the PKA Signaling, Stress Response, and Virulence in the Human Opportunistic Pathogen Aspergillus fumigatus
Source: Int J Mol Sci. 2019 Nov 11;20(22):5628. doi: 10.3390/ijms20225628 (PMC6888639; doi:10.3390/ijms20225628)
Supplement: Supplementary file 1 [file ijms-20-05628-s001.zip › Fig. S1.pptx]

## Slide 1
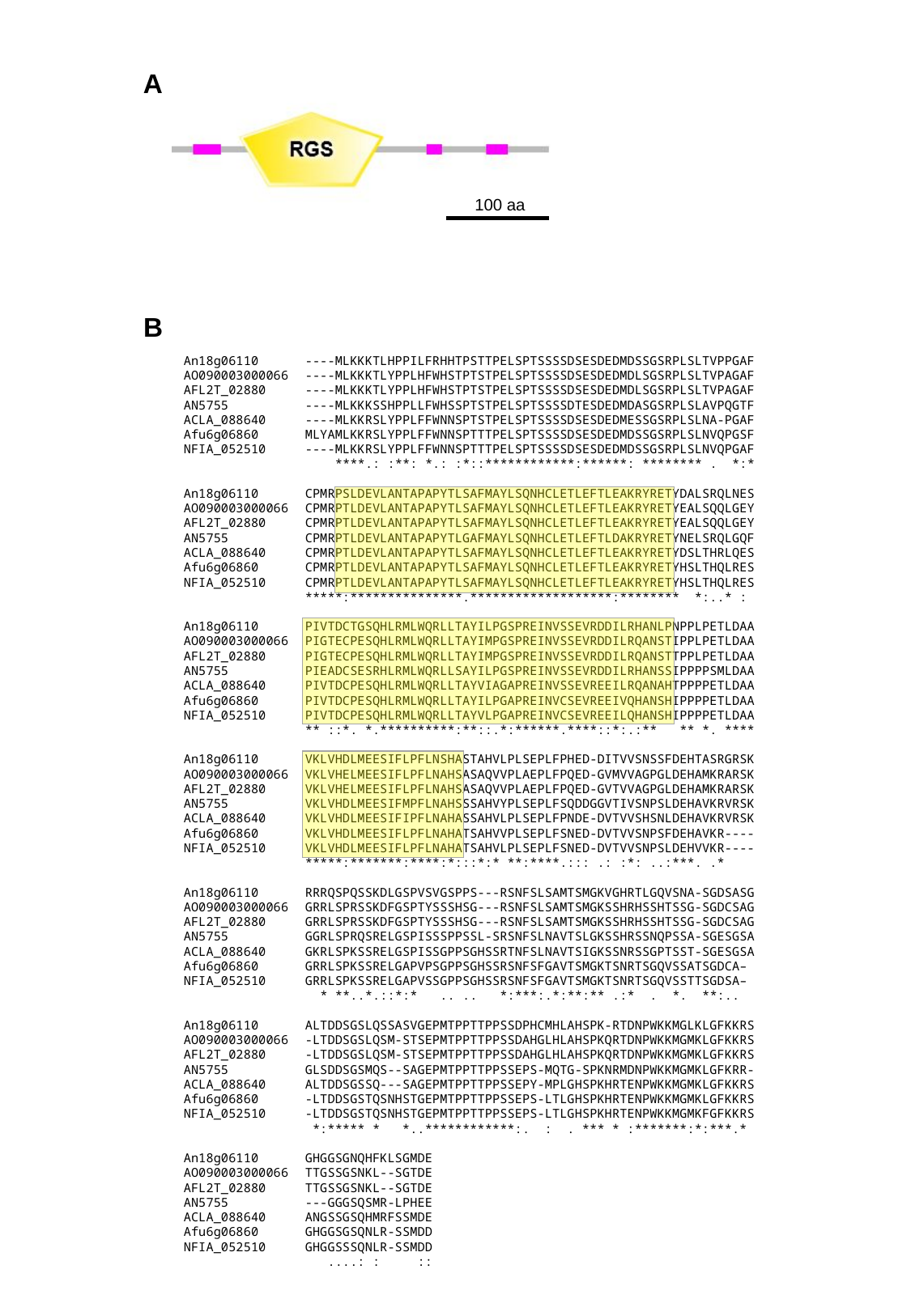

A
100 aa
B
An18g06110 	----MLKKKTLHPPILFRHHTPSTTPELSPTSSSSDSESDEDMDSSGSRPLSLTVPPGAF
AO090003000066 	----MLKKKTLYPPLHFWHSTPTSTPELSPTSSSSDSESDEDMDLSGSRPLSLTVPAGAF
AFL2T_02880 	----MLKKKTLYPPLHFWHSTPTSTPELSPTSSSSDSESDEDMDLSGSRPLSLTVPAGAF
AN5755 	----MLKKKSSHPPLLFWHSSPTSTPELSPTSSSSDTESDEDMDASGSRPLSLAVPQGTF
ACLA_088640 	----MLKKRSLYPPLFFWNNSPTSTPELSPTSSSSDSESDEDMESSGSRPLSLNA-PGAF
Afu6g06860 	MLYAMLKKRSLYPPLFFWNNSPTTTPELSPTSSSSDSESDEDMDSSGSRPLSLNVQPGSF
NFIA_052510 	----MLKKRSLYPPLFFWNNSPTTTPELSPTSSSSDSESDEDMDSSGSRPLSLNVQPGAF
	 ****.: :**: *.: :*::************:******: ******** . *:*
An18g06110 	CPMRPSLDEVLANTAPAPYTLSAFMAYLSQNHCLETLEFTLEAKRYRETYDALSRQLNES
AO090003000066 	CPMRPTLDEVLANTAPAPYTLSAFMAYLSQNHCLETLEFTLEAKRYRETYEALSQQLGEY
AFL2T_02880 	CPMRPTLDEVLANTAPAPYTLSAFMAYLSQNHCLETLEFTLEAKRYRETYEALSQQLGEY
AN5755 	CPMRPTLDEVLANTAPAPYTLGAFMAYLSQNHCLETLEFTLDAKRYRETYNELSRQLGQF
ACLA_088640 	CPMRPTLDEVLANTAPAPYTLSAFMAYLSQNHCLETLEFTLEAKRYRETYDSLTHRLQES
Afu6g06860 	CPMRPTLDEVLANTAPAPYTLSAFMAYLSQNHCLETLEFTLEAKRYRETYHSLTHQLRES
NFIA_052510 	CPMRPTLDEVLANTAPAPYTLSAFMAYLSQNHCLETLEFTLEAKRYRETYHSLTHQLRES
	*****:***************.*******************:******** *:..* :
An18g06110 	PIVTDCTGSQHLRMLWQRLLTAYILPGSPREINVSSEVRDDILRHANLPNPPLPETLDAA
AO090003000066 	PIGTECPESQHLRMLWQRLLTAYIMPGSPREINVSSEVRDDILRQANSTIPPLPETLDAA
AFL2T_02880 	PIGTECPESQHLRMLWQRLLTAYIMPGSPREINVSSEVRDDILRQANSTTPPLPETLDAA
AN5755 	PIEADCSESRHLRMLWQRLLSAYILPGSPREINVSSEVRDDILRHANSSIPPPPSMLDAA
ACLA_088640 	PIVTDCPESQHLRMLWQRLLTAYVIAGAPREINVSSEVREEILRQANAHTPPPPETLDAA
Afu6g06860 	PIVTDCPESQHLRMLWQRLLTAYILPGAPREINVCSEVREEIVQHANSHIPPPPETLDAA
NFIA_052510 	PIVTDCPESQHLRMLWQRLLTAYVLPGAPREINVCSEVREEILQHANSHIPPPPETLDAA
	** ::*. *.**********:**::.*:******.****::*:.:** ** *. ****
An18g06110 	VKLVHDLMEESIFLPFLNSHASTAHVLPLSEPLFPHED-DITVVSNSSFDEHTASRGRSK
AO090003000066 	VKLVHELMEESIFLPFLNAHSASAQVVPLAEPLFPQED-GVMVVAGPGLDEHAMKRARSK
AFL2T_02880 	VKLVHELMEESIFLPFLNAHSASAQVVPLAEPLFPQED-GVTVVAGPGLDEHAMKRARSK
AN5755 	VKLVHDLMEESIFMPFLNAHSSSAHVYPLSEPLFSQDDGGVTIVSNPSLDEHAVKRVRSK
ACLA_088640 	VKLVHDLMEESIFIPFLNAHASSAHVLPLSEPLFPNDE-DVTVVSHSNLDEHAVKRVRSK
Afu6g06860 	VKLVHDLMEESIFLPFLNAHATSAHVVPLSEPLFSNED-DVTVVSNPSFDEHAVKR----
NFIA_052510 	VKLVHDLMEESIFLPFLNAHATSAHVLPLSEPLFSNED-DVTVVSNPSLDEHVVKR----
	*****:*******:****:*:::*:* **:****.::: .: :*: ..:***. .*
An18g06110 	RRRQSPQSSKDLGSPVSVGSPPS---RSNFSLSAMTSMGKVGHRTLGQVSNA-SGDSASG
AO090003000066 	GRRLSPRSSKDFGSPTYSSSHSG---RSNFSLSAMTSMGKSSHRHSSHTSSG-SGDCSAG
AFL2T_02880 	GRRLSPRSSKDFGSPTYSSSHSG---RSNFSLSAMTSMGKSSHRHSSHTSSG-SGDCSAG
AN5755 	GGRLSPRQSRELGSPISSSPPSSL-SRSNFSLNAVTSLGKSSHRSSNQPSSA-SGESGSA
ACLA_088640 	GKRLSPKSSRELGSPISSGPPSGHSSRTNFSLNAVTSIGKSSNRSSGPTSST-SGESGSA
Afu6g06860 	GRRLSPKSSRELGAPVPSGPPSGHSSRSNFSFGAVTSMGKTSNRTSGQVSSATSGDCA–
NFIA_052510 	GRRLSPKSSRELGAPVSSGPPSGHSSRSNFSFGAVTSMGKTSNRTSGQVSSTTSGDSA–
	 * **..*.::*:* .. .. *:***:.*:**:** .:* . *. **:..
An18g06110 	ALTDDSGSLQSSASVGEPMTPPTTPPSSDPHCMHLAHSPK-RTDNPWKKMGLKLGFKKRS
AO090003000066 	-LTDDSGSLQSM-STSEPMTPPTTPPSSDAHGLHLAHSPKQRTDNPWKKMGMKLGFKKRS
AFL2T_02880 	-LTDDSGSLQSM-STSEPMTPPTTPPSSDAHGLHLAHSPKQRTDNPWKKMGMKLGFKKRS
AN5755 	GLSDDSGSMQS--SAGEPMTPPTTPPSSEPS-MQTG-SPKNRMDNPWKKMGMKLGFKRR-
ACLA_088640 	ALTDDSGSSQ---SAGEPMTPPTTPPSSEPY-MPLGHSPKHRTENPWKKMGMKLGFKKRS
Afu6g06860 	-LTDDSGSTQSNHSTGEPMTPPTTPPSSEPS-LTLGHSPKHRTENPWKKMGMKLGFKKRS
NFIA_052510 	-LTDDSGSTQSNHSTGEPMTPPTTPPSSEPS-LTLGHSPKHRTENPWKKMGMKFGFKKRS
	 *:***** * *..************:. : . *** * :*******:*:***.*
An18g06110 	GHGGSGNQHFKLSGMDE
AO090003000066 	TTGSSGSNKL--SGTDE
AFL2T_02880 	TTGSSGSNKL--SGTDE
AN5755 	---GGGSQSMR-LPHEE
ACLA_088640 	ANGSSGSQHMRFSSMDE
Afu6g06860 	GHGGSGSQNLR-SSMDD
NFIA_052510 	GHGGSSSQNLR-SSMDD
	 ....: : ::
