# Supplementary material for: RgsA Attenuates the PKA Signaling, Stress Response, and Virulence in the Human Opportunistic Pathogen Aspergillus fumigatus
Source: Int J Mol Sci. 2019 Nov 11;20(22):5628. doi: 10.3390/ijms20225628 (PMC6888639; doi:10.3390/ijms20225628)
Supplement: Supplementary file 1 [file ijms-20-05628-s001.zip › Fig. S2.pptx]

## Slide 1
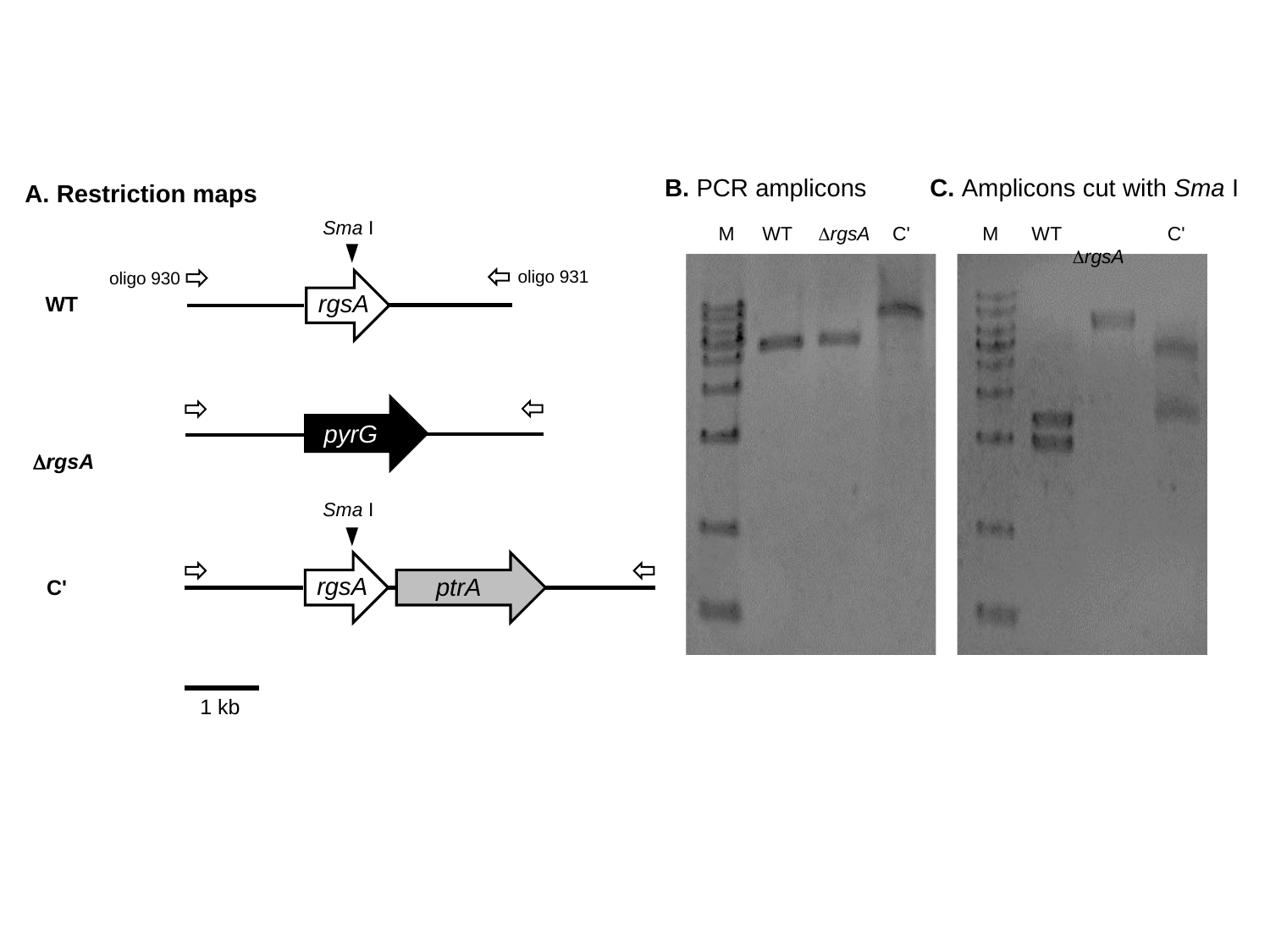

B. PCR amplicons C. Amplicons cut with Sma I
A. Restriction maps
Sma I
oligo 931
oligo 930
rgsA
pyrG
Sma I
rgsA
ptrA
1 kb
M WT
 DrgsA
C'
M WT
 DrgsA
 C'
WT
 DrgsA
 C'
