# Supplementary material for: RgsA Attenuates the PKA Signaling, Stress Response, and Virulence in the Human Opportunistic Pathogen Aspergillus fumigatus
Source: Int J Mol Sci. 2019 Nov 11;20(22):5628. doi: 10.3390/ijms20225628 (PMC6888639; doi:10.3390/ijms20225628)
Supplement: Supplementary file 1 [file ijms-20-05628-s001.zip › Supplementary Table 1.doc]

**Supplementary Table 1. Oligonucleotides used in this study.**

| **Name** | **Sequence (5'→3')^a^** | **Purpose** |
| --- | --- | --- |
| oligo346  oligo347  oligo303  oligo304  oligo305  oligo306  oligo256  oligo257  oligo430  oligo431  oligo432  oligo433  oligo434  oligo435  oligo508  oligo509  oligo514  oligo515  oligo1310  oligo1311  oligo1312  oligo1313  oligo1376  oligo1377  oligo1326  oligo1327  oligo697  oligo698  oligo926  oligo927  oligo928  oligo929  oligo930  oligo931  oligo935  oligo935  oligo936  oligo730  oligo731 | CCATGTGTGTCGAGTCCTTC  GAACGTACAGCAACAGTCTGG  GCTACCACTCTGCATCCTCA  TACGAGCTCCAGCATGATTC  ACGGCAGGAAGTTGTCTTCT'  CTGTCAGCGACTTGTTGGAT  TTCCAAGCAGAGCTTGTCAC  CCAGGTTCTTTGCACTTGAA  TTCCAAATGTGGCAAGTGAT  GCAAACGTGGAATCAATACG  GACCCAGCTAGGAACCACAT  AGTGACACCAAGACCCTTCC  CCACCACCTACAACAACAGC  TGTGAAGACGCATGATGAGA  GAGAATACCCTGCGGTTTGT  GGATCCTCTTCTTGGGATCA  ACTCCGAGGATGAGGATGAC  GACCCTGTTCCTTTCCAAGA  GGGCAAGAATCGGTCGCAGAAGTT  CGCAGGGATGGCACCGAAGTAAGA  CGCGGCGATACTTCCAGGTT  CATAATCGCCCACTCGTTGTCTA  CGATCGGGCGCAAACTCACG  TCCCCCTCCCGCGATTGTAACTTC  GAAAGGTCAGGTGTTCGAGTCA  CTTGGTTGCGGGTTTAGGGATT  GCAATGTAAAGCTAACGTGCGTG  TGCCTTTAAGCTTCGGGTAGAG  GTGCCCAAGTCTCAGCCACT  *TTTGTAGGCTTTGGGCTGTTCACAA*GCGGCACCCTTTTGGACTTG  *CTGATCTACCCCTTGGAACGCAGCA*TCCCTCTCTATCCGGCGGA  TGGCTGCGGGATCCTCAAAA  GACCAGTGGCGAGCACGATT  AGCTTCGACGTTGCGGAGTC  GCACAACCTGGGAGGACGAT  *AAACAAAGATGCAAGAGCGGC*TCCGCCGGATAGAGAGGGA  *CCAATGGGATCCCGTAATCAATT*GCATACCGTCACGCTGCATG  GCCGCTCTTGCATCTTTGTTT  AATTGATTACGGGATCCCATTGG | 5' *ef1* for qRT-PCR normalization  3' *ef1* for qRT-PCR normalization  5' *abaA* for qRT-PCR  3' *abaA* for qRT-PCR  5' *wetA* for qRT-PCR  3' *wetA* for qRT-PCR  5' *brlA* for qRT-PCR  3' *brlA* for qRT-PCR  5' *acyA* for qRT-PCR  3' *acyA* for qRT-PCR  5' *pksP* for qRT-PCR  3' *pksP* for qRT-PCR  5' *pkaC* for qRT-PCR  3' *pkaC* for qRT-PCR  5' *sakA* for qRT-PCR  3' *sakA* for qRT-PCR  5' *atfA* for qRT-PCR  3' *atfA* for qRT-PCR  5' *pbs2* for qRT-PCR  3' *pbs2* for qRT-PCR  5' *sskB* for qRT-PCR  3' *sskB* for qRT-PCR  5' *ssk1* for qRT-PCR  3' *ssk1* for qRT-PCR  5' for *A*. *fumigatus*-specific DNA qRT-PCR  3' for *A*. *fumigatus*-specific DNA qRT-PCR  5' *AnpyrG* marker  3' *AnpyrG* marker  5' flanking region of *rgsA*  5' *rgsA* with *AnpyrG* tail  3' *rgsA* with *AnpyrG* tail  3' flanking region of *rgsA*  5' nested of *rgsA*  3' nested of *rgsA*  5' *rgsA* for complementation  5' *rgsA* with *ptrA* tail for complementation  3' rgsA with *ptrA* tail for complementation  5' of *ptrA*  3' of *ptrA* |

^a^ Tail sequence is in italic.
